# An Ontology-Based Decision Support System for Tailored Clinical Nutrition Recommendations for Patients With Chronic Obstructive Pulmonary Disease: Development and Acceptability Study

**DOI:** 10.2196/50980

**Published:** 2024-06-26

**Authors:** Daniele Spoladore, Vera Colombo, Alessia Fumagalli, Martina Tosi, Erna Cecilia Lorenzini, Marco Sacco

**Affiliations:** 1 Institute of Intelligent Industrial Technologies and Systems for Advanced Manufacturing National Research Council of Italy Lecco Italy; 2 Department of Pure and Applied Sciences, Computer Science Division Insubria University Varese Italy; 3 Unit of Pulmonary Rehabilitation IRCCS, Italian National Research Center on Aging Casatenovo Italy; 4 Institute of Agricultural Biology and Biotechnology National Research Council of Italy Milan Italy; 5 Department of Health Science University of Milan Milan Italy; 6 Department of Biomedical Sciences for Health, Chair of Clinical Pathology University of Milan Milan Italy

**Keywords:** ontology-based decision support system, nutritional recommendation, chronic obstructive pulmonary disease, clinical decision support system, pulmonary rehabilitation

## Abstract

**Background:**

Chronic obstructive pulmonary disease (COPD) is a chronic condition among the main causes of morbidity and mortality worldwide, representing a burden on health care systems. Scientific literature highlights that nutrition is pivotal in respiratory inflammatory processes connected to COPD, including exacerbations. Patients with COPD have an increased risk of developing nutrition-related comorbidities, such as diabetes, cardiovascular diseases, and malnutrition. Moreover, these patients often manifest sarcopenia and cachexia. Therefore, an adequate nutritional assessment and therapy are essential to help individuals with COPD in managing the progress of the disease. However, the role of nutrition in pulmonary rehabilitation (PR) programs is often underestimated due to a lack of resources and dedicated services, mostly because pneumologists may lack the specialized training for such a discipline.

**Objective:**

This work proposes a novel knowledge-based decision support system to support pneumologists in considering nutritional aspects in PR. The system provides clinicians with patient-tailored dietary recommendations leveraging expert knowledge.

**Methods:**

The expert knowledge—acquired from experts and clinical literature—was formalized in domain ontologies and rules, which were developed leveraging the support of Italian clinicians with expertise in the rehabilitation of patients with COPD. Thus, by following an agile ontology engineering methodology, the relevant formal ontologies were developed to act as a backbone for an application targeted at pneumologists. The recommendations provided by the decision support system were validated by a group of nutrition experts, whereas the acceptability of such an application in the context of PR was evaluated by pneumologists.

**Results:**

A total of 7 dieticians (mean age 46.60, SD 13.35 years) were interviewed to assess their level of agreement with the decision support system’s recommendations by evaluating 5 patients’ health conditions. The preliminary results indicate that the system performed more than adequately (with an overall average score of 4.23, SD 0.52 out of 5 points), providing meaningful and safe recommendations in compliance with clinical practice. With regard to the acceptability of the system by lung specialists (mean age 44.71, SD 11.94 years), the usefulness and relevance of the proposed solution were extremely positive—the scores on each of the perceived usefulness subscales of the technology acceptance model 3 were 4.86 (SD 0.38) out of 5 points, whereas the score on the intention to use subscale was 4.14 (SD 0.38) out of 5 points.

**Conclusions:**

Although designed for the Italian clinical context, the proposed system can be adapted for any other national clinical context by modifying the domain ontologies, thus providing a multidisciplinary approach to the management of patients with COPD.

## Introduction

### Background

Chronic obstructive pulmonary disease (COPD) is one of the leading causes of morbidity and mortality worldwide. In Italy, 3.2% of the population in 2019 had a diagnosis of COPD, and such numbers are expected to increase in the next years due to the worsening of risk factors [1]. COPD is characterized by chronic inflammation in the lungs and airflow obstruction. Starting from the respiratory system, COPD has several systemic effects, including skeletal muscle wasting (which limits exercise capacity), increased risk of cardiovascular disease, osteoporosis, depression, and anxiety [2]. Pulmonary rehabilitation (PR) is an evidence-based, nonpharmacological intervention that helps people with COPD improve their health condition and quality of life by increasing exercise capacity and reducing symptoms of dyspnea and fatigue [3]. PR is based on a multidisciplinary approach and includes several components, among which nutritional status evaluation is considered relevant.

However, as highlighted recently by the American Thoracic Society, the scarcity of resources often limits the nutritional component, which, in some settings, can be accessible only outside the PR program [4]. Moreover, even though the influence of nutrition on COPD has been recognized and a relationship between some nutrients and pulmonary functions has been identified, PR programs often do not formally include patient-specific dietary recommendations. Therefore, more studies are required to drive the implementation of tailored nutritional strategies [5].

### The Role of Nutrition in Patients With COPD: Evidence

The relationship between COPD and nutrition has been investigated by many authors, who have put together a set of evidence that underlines the role of nutrients in PR and the disease exacerbations. The results of the systematic review and meta-analysis by Collins et al [6] demonstrated that nutritional support, primarily in the form of oral nutritional supplements, improves total energy intake, anthropometric measurements, and grip strength in patients with COPD. This work highlighted how dietary changes supported protein and energy intake in patients with COPD, resulting in weight gain and a moderate increase in muscle strength—which can significantly impact respiratory muscle strength, walking distance, and quality of life. Another systematic review [7] found that nutritional supplementation improves body weight gain in patients with COPD, especially when malnourished, in terms of body composition, respiratory muscle strength, and quality of life.

Inadequate nutritional intake, higher daily energy expenditure, weight loss, and fat-free mass (FFM) depletion affect the functional capacity of patients with COPD. The progression of the disease also contributes to an increased respiratory muscle load, hypoxemia, physical inactivity, and release of inflammation mediators [8]. In addition, Liu et al [9] explored the associations among COPD, diet, and inflammation, identifying a correlation between diet and COPD inflammatory status. By calculating the Diet Inflammatory Index in COPD, they observed that COPD was more prevalent in patients with a worse Diet Inflammatory Index.

A systematic review of factors influencing the risk of developing COPD [10] concluded that the Western diet, characterized by the consumption of processed meat and alcohol and a low intake of fruit and vegetables, is associated with the prevalence of COPD. A cross-sectional study [11] assessed the nutritional status of outpatients with COPD and found a significant association between malnutrition and COPD—most of the patients did not have an adequate daily food intake and showed a lower quality of life, highlighting the need for a more tailored nutritional intervention for managing malnutrition in patients with COPD with cachexia. In COPD, low FFM and sarcopenia are predictive factors of mortality [12]. Considering that literature data demonstrate that the presence of malnutrition and, above all, a low FFM index is associated with an increased risk of mortality, Schols et al [13] identified different nutritional risk profiles based on nutritional phenotypes that appear to be predictors of outcome independent of impaired respiratory function. These nutritional phenotypes included obesity, sarcopenic obesity, sarcopenia, and cachexia and required at least three items for their identification: (1) BMI and body circumference, (2) bioelectrical impedance analysis, and (3) history of unintentional weight loss. A recent study [14] demonstrated that patients with COPD with sarcopenia and sarcopenic obesity had worse muscle strength than patients with healthy body weight, claiming that body composition is associated with physical functions. Patients with COPD with obesity have a higher risk of developing comorbidities such as diabetes and metabolic and cardiovascular diseases in addition to typical COPD symptoms.

### Research Challenge: Nutritional Status to Prevent COPD Exacerbation

The evidence reported previously indicates that clinicians and dietitians must evaluate the nutritional status and body composition of patients when giving nutritional advice or diet recommendations to patients with COPD with obesity to foster an adequate intake of macro- and micronutrients and control obesity as well [15].

Malnutrition plays a pivotal role among the challenges that must be faced in treating COPD. The study by Mete et al [16] affirms that malnutrition and risk of malnutrition are very frequent conditions among patients with COPD and, together with a significantly lower BMI, are associated with disease severity, as highlighted by pulmonary function tests. In clinical practice, sarcopenia assessment is not part of the standard of care, implying a loss of personalized treatment that should be essential to improve health outcomes related to the disease [17]. The current literature illustrates that screening for sarcopenia and tailored nutrition intervention in patients with COPD could cost-effectively achieve better health outcomes [18,19]. Functional capacity, respiratory muscle strength, and quality of life can all be improved through nutritional supplementation and a better assessment of nutritional status.

The future nutrition challenges require the identification of specific targets of intervention, considering body composition, nutritional status, and inflammation in addition to the strictly clinical aspects. Nowadays, diet is not always an integral part of COPD therapeutic strategy [20]. Moreover, different studies have highlighted the necessity of new methods to assess malnutrition and evaluate nutritional status in patients with COPD, not considering the BMI alone [21] as it misses important changes in body composition.

This new vision places nutritional intervention as an integral part of COPD management, not only in the advanced and early stages but also to prevent the evolution toward respiratory failure. Creating a decision support system (DSS) that brings together data and knowledge in the nutritional field to identify the different nutritional phenotypes and suggest specific dietary recommendations could be useful to meet these challenges. In particular, this DSS could become a valuable tool for pulmonologists to develop nutritional interventions as an integral part of the COPD therapeutic strategy even in the absence of specific resources.

### Objective of This Study

Digital health care applications are already used to improve PR models, mainly for self-management; modification of lifestyle factors; and modification of risk factors, such as smoking cessation and fostering physical activity [22]. However, to the best of our knowledge, there are only a few applications focused on nutritional aspects associated with COPD. This work leveraged clinical expert knowledge—formalized into domain ontologies—from the Italian health care context to develop a DSS to support pneumologists in considering nutritional aspects in PR to avoid the disease’s exacerbation and provide patients with tailored dietary guidelines. The application exploiting the DSS fits the context of a preprototype according to the World Health Organization (WHO) guidelines for the monitoring and evaluation of digital health interventions [23].

### Related Work

#### Overview

Ontology-based technologies have been adopted for both clinical DSSs and patient-centered DSSs. Regarding the ontological formalization of requisites and tailored suggestions for patients with COPD in DSSs, no work has tackled this issue in PR—except for an early version of the system we propose in this work [24]. Nonetheless, the use of ontologies to formalize COPD has been established in some works. Moreover, specific digital applications for patients with COPD have been developed in the past years. This section presents works relevant to the fields of ontology-based clinical and patient-centered DSSs and digital applications specifically developed for patients with COPD.

#### Ontologies and Ontology-Based Systems for COPD Management

COPD has been formalized in ontologies or knowledge bases since the early 2010s. The COPD ontology by Greenberg et al [25] was developed to support COPD longitudinal research and clinical trials, leveraging a preexisting model dedicated to representing subpopulations of patients. Cano et al [26] developed a knowledge base devoted to collecting clinical experimental data; the COPD Knowledge Base can semantically map data to physiological and molecular data to support clinical decision-making through a predictive mathematical model.

Ontologies can play a pivotal role in diagnostic systems. In the case of COPD, Rayner et al [27] developed an ontology for the early diagnosis of the illness. The ontological model takes advantage of clinical tests and patients’ data (eg, spirometry, forced expiratory volume in the first second [FEV1], age, and smoking status) and tests its robustness against a large data set of patients. The model proved able to categorize patients as “Unlikely COPD,” “Probable COPD,” and “Definite COPD” cases.

As ontology-enabled reasoning processes are appreciated in the context of clinical decision-making, the adoption of formal models in health care systems aimed at managing chronic conditions—including systems for patients with COPD—has also been established. CHRONIOUS [28] is an open and ubiquitous system that exploits ontology-based inferential reasoning to adapt the platform’s services, including monitoring patients’ conditions—which is also performed leveraging wearable sensors. Lasierra et al [29] developed an ontology-based telemonitoring system aimed at monitoring patients with chronic diseases at home. The ontology layer (Home Ontology for Integrated Management in Home-Based Scenarios) represents the patient profile, vital signs, and chronic conditions (including COPD) to observe the patient’s evolution and plan activities. Similarly, Ajami and McHeick [30] developed a domain ontology encompassing environmental features, patient data, clinical status and diseases (including COPD), and devices to monitor and identify patients’ conditions. This DSS aimed to foster patients’ adherence to a healthy lifestyle and identify and avoid possibly dangerous situations.

#### Digital Applications for Patients With COPD

Digital applications may offer solutions to both clinicians—to ease the process of assessment and support clinical decisions—and patients—mainly to support them in the management of the disease. Digital applications currently available for patients with COPD are mainly telemedicine and telerehabilitation solutions. Most of them are focused on educational programs, symptom tracking, behavior change, support for medication or treatment, and activity report [31]. In some cases, as both research prototypes [32] and commercial products [33], the educational programs include specific sections on nutrition, in which helpful tips and recommendations are provided to manage symptoms during meal consumption and help maintain a balanced diet. However, such applications do not provide personalized information tailored to the patient’s nutritional status. In such cases, the applications targeted at health care professionals comprise the “clinician side” of telemedicine platforms (ie, monitoring dashboard allowing for remote visualization of patients’ data and applications for remote teleconsulting).

Differently from the aforementioned solutions, the DSS proposed in this work tackles the role of nutritional therapy in the PR of patients with COPD—an aspect neglected in existing DSSs.

## Methods

A DSS was developed to tackle the aims described in the Introduction section and the research challenge described in the Objective of This Study section, thus supporting pneumologists in suggesting specific dietary recommendations for patients with COPD. The DSS leverages expert knowledge in the form of ontologies and, through automatic inference processes, is expected to provide guidelines to prepare a tailored dietary plan.

The development of the ontology underlying the DSS leveraged expert clinical knowledge to maximize its acceptability. Contrary to purely data-driven approaches, ontologies formalize information to enable a system to perform inferences (based on the knowledge formalized in the ontology). The inference process simulates human inference capabilities [34] so that ontology-based approaches are perceived as transparent. Thus, ontologies are widely adopted in several artificial intelligence and health-related applications [35].

However, the development of a domain ontology is not a trivial task—it is a process that may involve several activities (eg, the acquisition of knowledge, its conceptualization, the survey of existing models that can be reused, the development of the model in a formal language, and the testing of the developed ontology [36]). The ontology engineered for the proposed DSS was developed following the Agile, Simplified, and Collaborative Ontology Engineering Methodology (AgiSCOnt) [37] engineering methodology, which involves knowledge elicitation techniques and domain experts in the development phase of the ontology. This collaborative ontology engineering methodology adopts unstructured interviews, scientific literature surveys, and discussions to elicit the necessary knowledge to minimize the impact of the “knowledge elicitation bottleneck” (ie, it takes longer to gather knowledge from experts and documentation than to write the software [38]). Its collaborative and agile features and validation [37] were the reasons behind the adoption of AgiSCOnt in this work.

The methodology involves three phases:

1. *Domain analysis and conceptualization*, which includes the identification of the knowledge to be included and the preparation of competency questions (CQs) [39] that the ontology is expected to answer; it enables the conceptualization of the domain (which results in a conceptual map) and the preliminary identification of existing ontological resources that can be reused.
2. *Development and testing*, which involves the selection of the ontological languages to formalize the conceptualization developed in the previous step and the identification of ontology design patterns (ie, “micro-ontologies” that can be reused to model recurrent problems in ontology engineering [40]). This step results in the prototypization of the ontology, which undergoes a preliminary test to assess the validity of its inferences.
3. *Ontology use and updating*, which includes activities such as use of the developed ontology in an application, extended validation, and feedback collection. The following subsections delve into the engineering process.

The development of this ontology involved the following team members: 1 ontologist with experience in modeling using agile ontology engineering methodologies, 1 biomedical engineer with previous experience in ontology engineering and knowledge of COPD, 2 senior dieticians with clinical experience with patients with COPD, and 1 senior pneumologist. The team was composed of clinical personnel from universities (dieticians) and a research and cure center (Scientific Institute for Research, Hospitalisation and Health Care) with a specialization in COPD (pneumologist) and yearly experience treating such patients. The team delved into nutrition and diet’s role in tackling this disease, with examples from the literature and clinical trials in which the clinical personnel was involved. The discussion was then oriented to identify some of the issues that the ontology was expected to answer (ie, the CQs).

### Ethical Considerations

This study does not include human subjects research (no human subjects experimentation or intervention was conducted) and so does not require institutional review board approval.

## Results

This section describes the development of the COPD and Nutrition domain ontology for the DSS and the ontology-based application for clinical personnel.

### The COPD and Nutrition Domain Ontology

#### Domain Analysis and Conceptualization

The considerations reported in The Role of Nutrition in Patients With COPD: Evidence and Research Challenge: Nutritional Status to Prevent COPD Exacerbation sections were gathered by the team in this phase. Leveraging the objective (ie, providing clinical personnel with support in identifying tailored nutritional recommendations for patients with COPD), the team decided that the ontology should focus on representing the patients’ health condition and the stage of their COPD. On the basis of their expertise, clinicians pointed out that the purpose of the ontology should be to illustrate, for each patient, a tailored percentage of macro- and micronutrients they are advised to consume on a daily basis to avoid exacerbations, as well as to provide nutritional guidance. The summary of the entities and expected outputs of the ontology is provided in Textbox 1, listing the CQs and their answers.

**The list of competency questions and answers for the Chronic Obstructive Pulmonary Disease (COPD) and Nutrition ontology engineering process.**

**What information identifies a patient? What basic information is used to identify the patient? What clinical information is used to identify the patient?**

- A patient is identified by an ID and gender. Each patient is associated with 1 health condition and 1 anthropometric phenotype (defined via BMI cutoffs). Each patient can be classified as a patient with sarcopenia or cachexia or as a patient without sarcopenia or cachexia.

**How is COPD evaluated?**

- COPD is evaluated according to the criteria defined in the gold standard—it can be mild, moderate, severe, and very severe. The criterion to be analyzed is the forced expiratory volume in the first second.

**How is sarcopenia evaluated?**

- The status of sarcopenia is evaluated according to clinical standards (operational definition of sarcopenia). The first criterion comprises low muscle strength, the second criterion comprises a low muscle quantity or quality, and the third criterion comprises low physical performance. The presence of the first criterion alone indicates probable sarcopenia, the presence of both the first and second criteria indicates diagnosed sarcopenia, and the presence of all 3 criteria indicates severe sarcopenia.

**How is cachexia evaluated?**

- Cachexia is evaluated by means of biochemical indicators according to the study by Evans et al [41]. Albuminemia, iron transport, and polymerase chain reaction (PCR) criteria must be copresent to indicate a cachexia diagnosis.

**Which data characterize the patient’s health condition? How is the nutritional risk index assessed?**

- Patients’ health condition must indicate the stage of COPD and the nutritional risk index profile characterizing the patient. Each health condition must illustrate anthropometric measures (current weight, usual weight, height in meters, and BMI), physical performance indicators (hand grip and gait speed), and biochemical indicators (albuminemia, PCR, resistance, reactance, and iron transport). The nutritional risk index assessment is performed following clinical standards.

**What recommendations are given to clinical personnel?**

- The recommendations provided to clinical personnel indicate (for each patient) the basal metabolic rate and the corrected caloric intake, the daily macronutrient shares (protein, minimum and maximum share of carbohydrates, minimum and maximum share of fats, minimum and maximum share of fiber, maximum share of sugar, and maximum share of saturated fats), the amount of cholesterol and sodium, and whether the patient should increase their caloric intake by means of branched-chain amino acid or energy-protein supplementations.

**How are recommendation values calculated?**

- The indications provided in the patient’s recommendation are calculated according to clinical standards and differentiated according to the patient’s gender, stage of COPD, and anthropometric phenotype.

In this phase, the pneumologist specialized in clinical nutrition and a clinical dietitian defined the phenotypes and their nutritional requirements based on anthropometric and clinical parameters and comorbidities according to national and international guidelines. For the definition of each anthropometric phenotype, COPD stage (defined via FEV1 and forced vital capacity and in particular FEV1–to–forced vital capacity ratio [42,43]; Table 1) and the presence of sarcopenia or cachexia were considered. A total of 5 metabolic phenotypes for patients with COPD were developed (underweight, normal weight, overweight, first-degree obesity, and second-degree obesity), and each can be characterized by the presence of sarcopenia, cachexia, or none of the 2 conditions (Table 2).

Moreover, nutritional risk was assessed by means of the nutritional risk index (NRI) formula [44] as follows:



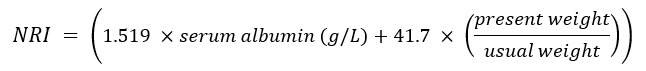



**(1)**

Thus, patients were classified according to this formula (Table 3).

The diagnosis of sarcopenia was based on the analysis of specific patients’ value indicators. The first one was the appendicular skeletal muscular mass, which is calculated according to the work by Sergi et al [45]:



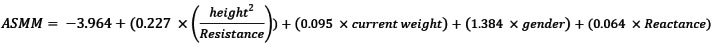



**(2)**

Appendicular skeletal muscular mass and other indicators allow for the classification of patients’ sarcopenic condition according to the 3 criteria in Table 4 [46].

If the first criterion applied, then the patient was *probable* sarcopenic; if the first and second criteria applied, then the patient was *diagnosed* sarcopenic; if all 3 criteria applied, then the patient was *severe* sarcopenic.

A similar approach was adopted to identify whether a patient was cachectic. If a patient’s polymerase chain reaction was >10, the level of iron transport was <150, the level of albuminemia was <3.5, and the patient was sarcopenic, then they were also cachectic [41]. As such, it is safe to infer that cachexia is a particular case of sarcopenia.

Calculation of nutritional recommendations was performed using the “if-then” type rules produced by clinical personnel considering reference values reported in scientific literature and national and international guidelines. The COPD DSS provides the following nutritional information and recommendations: (1) basal metabolic rate (BMR), (2) total daily energy requirement (kcal), (3) meal frequency, (4) indication for energy-protein supplementation (yes or no), (5) indication for branched-chain amino acid (BCAA) supplementation (yes or no), (6) protein intake (percentage and grams), (7) carbohydrate intake (percentage and grams), (8) lipid intake (percentage and grams), (9) sugar intake (maximum percentage and grams), (10) saturated fat intake (maximum percentage and grams), (11) cholesterol intake (maximum milligrams), (12) fiber intake (minimum and maximum grams), (13) sodium intake (maximum milligrams), and (14) calcium intake (milligrams).

For BMR calculation, Harris-Benedict or Mifflin predictive equations based on sex, age, weight, and height were considered. The Harris-Benedict equation was preferred for patients who were underweight or had normal weight, whereas the Mifflin equation was used for patients with COPD with overweight or obesity [47-49]. According to the COPD stage, different correction factors to BMR were applied (Multimedia Appendix 1)—for patients who were underweight, had a normal weight, were overweight, or had first- or second-class obesity presenting with COPD at the first or second stage and who were nonsarcopenic and noncachectic or sarcopenic, a 1.5 correction factor was used to calculate total daily energy requirement; for the same categories of patients presenting with COPD at the third or fourth stage, a correction factor of 1.8 was preferred to counteract the important energy expenditure due to the respiratory work of these patients. Only for patients with cachexia a 1.8 correction factor was always applied regardless of BMI and COPD stage.

The impact of physical activity was deemed marginal for the correction factor’s definition as patients with COPD are a vulnerable population presenting with comorbidities that limit their capacity to perform physical activity. As seen in the pathological lung mechanics of patients with COPD, dynamic hyperinflation influences the proper operation of the chest’s horizontal and vertical diameters that expand during inspiration due to the activation of the external intercostal muscles and the diaphragmatic contraction. This impairment plays a role in how much exercise a patient with COPD can tolerate. As shown in individuals with severe-stage COPD and weight loss related to COPD, respiratory muscle weakness exacerbates the breathing mechanism. Increased dyspnea and decreased exercise tolerance are directly related to this respiratory muscle weakening. Moreover, it has been observed that patients with COPD are characterized by higher levels of physical inactivity [50]. As far as protein requirement was concerned, clinical experts decided to provide different recommendations according to real or ideal weight or BMI or focusing on FFM considering specific body composition or COPD stage to give personalized recommendations to not compromise health and nutritional status as well as prevent further decrease in metabolically active lean mass [12]. For this reason, cachectic phenotype, regardless of BMI and COPD stage, was always given a high percentage of protein intake. BCAA was suggested when energy requirements but not protein requirements were met through diet. Differently, protein-caloric supplements were indicated when neither energy nor protein requirements were satisfied through food intake [51]. Phenotypes characterized by cachexia, regardless of BMI, underweight sarcopenic and normal-weight phenotype, and COPD stage, were always recommended BCAA and energy-protein supplementation in consideration of their health status. For the same reason, BCAA supplementation was always suggested for sarcopenic phenotypes [52]. As far as carbohydrate metabolism was concerned, and in line with the lower levels reported in the reference values of nutrients and energy for the Italian population [13,47], a carbohydrate intake of 45% to 50% of total daily calories and a maximum sugar intake of 15% of total calories were indicated except for individuals with type 2 diabetes mellitus, for whom a maximum of 10% of total calories was indicated to be reached through sugar intake. A lower recommendation for carbohydrate intake permits the promotion of protein and lipid intake, which are functional for respiratory work and to prevent further weight or lean mass loss. Regarding dietary fiber, it was decided that providing a lower indication than that provided by the Livelli di Assunzione di Riferimento di Nutrienti ed energia (LARN; National Recommended Energy and Nutrient Intake Levels) was preferred to encourage the intake of energy and protein foods, especially considering the difficulties met by patients with COPD in feeding and the early sense of satiety as a result of high-fiber food intake. Compared to the LARN, a higher intake of lipids was recommended, especially for phenotypes presenting with a partial pressure of carbon dioxide of >50 mm Hg, a measure of carbon dioxide in arterial or venous blood [53]. For saturated fats, a maximum intake of 10% of total daily calories was suggested according to LARN guidelines. Regarding cholesterol intake, a LARN nutritional goal for prevention, a maximum of 300 mg per day was recommended except for patients with high cholesterol levels, for whom the target was lowered to 200 mg per day. Hypercholesterolemia was defined as elevated total or low-density lipoprotein cholesterol levels or low levels of high-density lipoprotein cholesterol. Regarding micronutrients, sodium and calcium intake was considered. According to the recent WHO report on sodium intake [54], a maximum intake of <2000 mg per day of sodium (<5 g per day of salt) was recommended. A slightly higher recommendation than that of the LARN was given for calcium to prevent or counteract osteoporosis [55]. Finally, given the difficulties in feeding and early satiety observed, an indication for fractioned meals was given to meet energy and nutritional requirements throughout the day with small and frequent meals.

**Figure 1:**
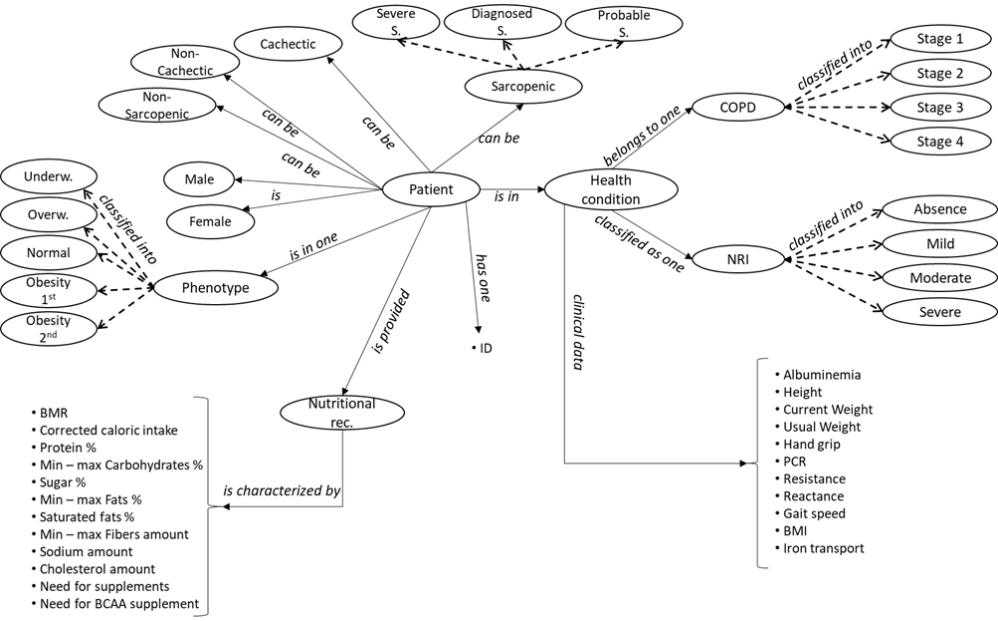
An excerpt of the conceptual map developed by the team involved in the ontology engineering process for the clinical chronic obstructive pulmonary disease (COPD) and nutrition ontology. BCAA: branched-chain amino acid; BMR: basal metabolic rate; NRI: nutritional risk index; PCR: polymerase chain reaction.

All the parameters involved in the evaluations presented previously (ie, FEV1; partial pressure of carbon dioxide; resistance; reactance; iron transport; albuminemia; polymerase chain reaction; hand grip; gait speed; and general patient information such as age, gender, height in meters, current weight, and usual weight) are usually acquired during patient assessment (such as spirometry and blood tests). AgiSCOnt’s outputs for the domain analysis phase consisted of the conceptual map reported in Figure 1 and a list of CQs (Textbox 1).

**Table 1:** The cutoffs identifying the chronic obstructive pulmonary disease (COPD) stages based on the forced expiratory volume in the first second (FEV1) values.

COPD stage number	COPD stage name	FEV1 (%)
I	Mild	≥80
II	Moderate	≥50 to <80
III	Severe	≥30 to <50
IV	Very severe	<30

**Table 2:** World Health Organization cutoffs for nutritional status categories based on BMI.

Nutritional status	BMI (kg/m^2^)
Underweight	<18.5
Healthy weight	≥18.5 to ≤24.9
Overweight (preobesity)	≥25.0 to <29.9
Obesity degree I	≥30.0 to <34.9
Obesity degree II	≥35.0 to <39.9
Obesity degree III	≥40

**Table 3:** The cutoffs identifying the 4 levels of nutritional risk based on the nutritional risk index (NRI).

NRI	Index
Absence of risk	>100
Mild risk	≥97.5 to ≤100
Moderate risk	≥83.5 to <97.5
Severe risk	<83.5

**Table 4:** Criteria for the classification of sarcopenia in patients.

Criteria	Cutoff for male patients	Cutoff for female patients
Low muscular strength—hand grip	<27 kg	<16 kg
Low muscular quantity	ASMM^a^<20 kg; ASMM/height^2^<7 kg/m^2^	ASMM<15 kg; ASMM/height^2^<5.5 kg/m^2^
Poor physical performance	≤0.8 m/s	≤0.8 m/s

^a^ASMM: appendicular skeletal muscular mass.

#### Development and Testing

The development phase adopted the conceptual map and CQs produced in the previous phase to guide the entire development process and discuss whether to model some concepts pertaining to the patients or their health conditions. The ontology editor Protégé (Stanford Center for Biomedical Informatics Research) [56] that supports Resource Description Framework [57] and Web Ontology Language [58] with a DL (description logic) profile [59] was adopted. Clinicians explicitly asked any significant advancement in the development of TBox and Abox (eg, patient modeling, health condition characterization, and recommendation modeling) to be illustrated to ensure that undesired entailments were not modeled in the ontology. From a reuse perspective, no ontology able to describe the conceptualization reached in this study was found; the only ontology design pattern reused in this ontology was the one that relates a copd:Patient to their copd:Health_Condition via the copd:isInHealthCondition object property [60]. The developed ontology (prefixed with copd:), discussed in this subsection, is accessible in Multimedia Appendix 2.

The development started with the identification of concepts that could be translated into owl:Classes. The concept of copd:Patient is pivotal in this ontology. Each patient is defined by exactly 1 patient ID, is given at least 1 nutritional recommendation individual, and is characterized by a health condition individual.

Each patient needs to be classified as copd:Female or copd:Male—disjoint classes—and as copd:Cachectic (or its complement copd:non-Cachectic) or copd:Sarcopenic (or its complement copd:non-Sarcopenic). Sarcopenia and cachexia are modeled as attributes of the patient and not of their health condition. The clinical personnel deemed essential to state that these 2 conditions have systemic status; therefore, they characterize the individual as a whole. The class copd:Sarcopenic is further detailed into the subclasses copd:Probable_Sarcopenic, copd:Diagnosed_Sarcopenic, and copd:Severe_Sarcopenic to reflect the operational definition standard provided by clinicians (presented in the previous subsection).

In the same way, the copd:Anthropometric_Phenotypes are characteristics of the copd:Patients, and this class lists 5 subclasses for the representation of the phenotypes.

Similarly to copd:Patient, the development of the TBox pertaining to the patient’s health condition was discussed among the team members—each health condition is characterized by an NRI profile, but in general, a copd:Health_Condition is not necessarily characterized by COPD. The terms adopted in the conceptual map to sketch the relationships among copd:Health_Condition, copd:Nutritional_Risk_Index_Profile, and copd:COPD_HC were found indicative of the clinicians’ perspective—both NRI and COPD are considered particular attributes of a health condition (ie, there could be health conditions characterized only by an NRI profile but lacking COPD). Therefore, the classes copd:Nutritional_Risk_Index_Profile and copd:COPD_HC were modeled as rdfs:subclassOf copd:Health_Condition. This decision was also encouraged by the fact that the datatype properties copd:FEV1 and copd:nutritionalRiskIndex have copd:Health_Condition as the domain.

The copd:Nutritional_Risk_Index_Profile and copd:COPD_HC subclasses are characterized by restrictions that allow for the classification of individual health conditions whose copd:nutritionalRiskIndex and copd:FEV1 object values fall under specific restrictions.

**Figure 2:**
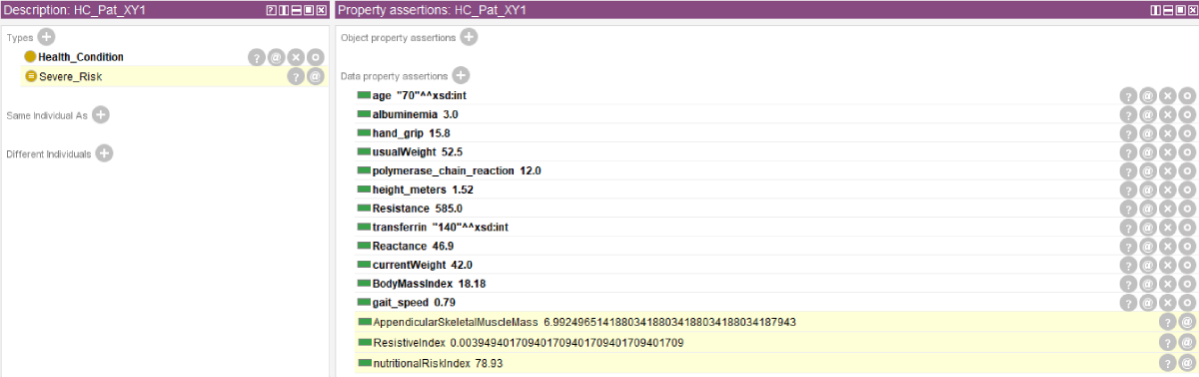
The complete datatype property set for a copd:Health_Condition. The datatype properties with a yellow background represent inferred values.

Each copd:Health_Condition is described by a set of datatype properties, which represent the clinical data elicited in the previous phase and are required to enable the patient’s classification and recommendations. Each owl:Individual belonging to this class also materializes inferred triples related to the copd:AppendicularSkeletalMuscleMass, the copd:ResistiveIndex, and the copd:nutritionRiskIndex. While the copd:nutritionalRiskIndex is calculated using semantic web rule language (SWRL) rules and used to classify each copd:Health_Condition into one of the NRI’s subclasses, the copd:ResistiveIndex is a piece of information necessary to calculate the copd:AppendicularSkeletalMuscleMass (both are inferred as the result of 2 different SWRL rules). Figure 2 illustrates an example of copd:Health_Condition completed with all its datatype properties (both asserted and inferred).

The ontology makes use of 39 datatype properties and 2 object properties (copd:isInHealthCondition and copd:hasRecommendation)—almost all datatype properties were used to provide values for the patient’s health condition and nutritional recommendation.

The ontology also contains 79 SWRL rules, which are largely used to represent the tuples in Multimedia Appendix 1, depicting the conditions that determine the shares and amounts of nutrients characterizing a patient’s diet (see the full ontology in Multimedia Appendix 2). The equations adopted to calculate the BMR and the corrected caloric intake were adapted with SWRL using mathematical built-ins [61]. Taking as an example a copd:Overweight, copd:non-Sarcopenic, and copd:non-Cachectic male patient characterized by copd:Stage2 disease, the BMR is inferred through the following rule (for each male patient not characterized by sarcopenia or cachexia, calculate the BMR using the Harris-Benedict equation and round the result):

Male(?p), Overweight(?p), (not (Cachectic))(?p), (not (Sarcopenic))(?p), hasRecommendation(?p, ?rec), isInHealthCondition(?p, ?hc), age(?hc, ?age), currentWeight(?hc, ?kg), height_meters(?hc, ?m), multiply(?a, ?kg, 13.75), multiply(?b, ?m, 5, ?100), multiply(?c, 6.78, ?age), add(?d, 66.5, ?a, ?b), subtract (?e, ?d, ?c), round(?f, ?e) -> regularRecommendedCaloricIntake(?rec, ?f)

Then, the correction is applied (for each male patient not characterized by sarcopenia or cachexia with stage-1 or stage-2 COPD, correct the BMR calculated using the Harris-Benedict equation by multiplying it by 1.5):

(Normal_Weight or Obesity_1st_Degree or Overweight or Underweight)(?p), hasRecommendation(?p, ?rec), isInHealthCondition(?p, ?hc), (Stage1 or Stage2)(?hc), (not (Cachectic))(?p), (not (Sarcopenic))(?p), regularRecommendedCaloricIntake(?rec, ?reg), multiply(?corin, ?reg, 1.5), round(?f, ?corin) -> correctedRecommendedCaloricIntake(?rec, ?f)

The definition of the share of protein that a patient with COPD needs is calculated by identifying the amount (in grams) of protein. With the sole exception of patients with cachexia—who are given a 25% protein share for clinical reasons—for each patient, the daily quantity of protein is calculated according to their weight (for each patient with normal or overweight status and not characterized by sarcopenia or cachexia, obtain the amount of protein in grams by multiplying their current weight by 1.2):

(Normal_Weight or Overweight)(?p), (not (Cachectic))(?p), (not (Sarcopenic))(?p), hasRecommendation(?p, ?rec), isInHealthCondition (?p, ?hc), currentWeight (?hc, ?w), multiply (?pgra, ?w, 1.2) -> proteinsGrams (?rec, ?pgra)

This amount is then converted into calories, bearing in mind that 1 protein is equal to 4 kcal, and then the daily protein share is calculated. This approach also enables the possibility to correct the amount of protein for particular classes of patients; for example, dieticians indicated that patients classified as copd:non-Sarcopenic, copd:non-Cachectic, and copd:Underweight should have their protein share calculated considering a different BMI (which is set to a higher value to fight their underweight condition and is established at 22.5 kg/m^2^).

As mentioned previously, the ontology provides enough SWRL rules to model all the information identified by the domain experts and elicited in Multimedia Appendix 1. As established by the development step in AgiSCOnt, the ontology underwent a test with data from 6 patients provided by clinicians. The test was divided into 2 steps. The first was dedicated to assessing whether the ontology provided a correct classification of the patients (ie, whether it identified copd:Sarcopenic and copd:Cachectic status for each patient and whether the stage of COPD and the copd:Nutritional_Risk_Index_Profile were correctly inferred). Thus, by querying the ontology using SPARQL (World Wide Web Consortium) [62], it was possible to assess the accuracy of the inferences (reported in Table 5).

The pneumologist and dieticians verified the correctness of the classification for each patient. All 6 individuals representing patients were found to be correctly classified. The second phase dealt with the retrieval of nutritional suggestions and their evaluation by the clinical personnel with the aim of assessing the validity of the SWRL rules modeled in the COPD and Nutrition ontology. The ontology was queried using SPARQL to retrieve all the nutrient minimum and maximum shares and quantities deemed important for patients with COPD (the results of the query are reported in Multimedia Appendix 3). Each copd:Nutritional_Recommendation and its inferred nutrient values were evaluated by clinical personnel and were found to be correct—although, for some values such as the copd:proteinShare and copd:fiberMINamount, a rounding of the decimal was suggested by dieticians.

**Table 5:** An excerpt of the results retrieved for the query investigating, for each patient, their ID, their status (whether they had sarcopenia or cachexia), the stage of chronic obstructive pulmonary disease (COPD), and their nutritional risk index profile. For patients characterized by sarcopenia, all the subclasses of copd:Sarcopenic are illustrated so that clinical personnel can easily see the importance of this condition.

?id	?status	?antrPhen	?copdstage	?nri
001	copd:Cachectic	copd:Underweight	copd:Stage1	copd:Severe_Risk
001	copd:Diagnosed_Sarcopenic	copd:Underweight	copd:Stage1	copd:Severe_Risk
001	copd:Female	copd:Underweight	copd:Stage1	copd:Severe_Risk
001	copd:Probable_Sarcopenic	copd:Underweight	copd:Stage1	copd:Severe_Risk
001	copd:Sarcopenic	copd:Underweight	copd:Stage1	copd:Severe_Risk
001	copd:Severe_Sarcopenic	copd:Underweight	copd:Stage1	copd:Severe_Risk
...^a^	...	...	...	...
BB	copd:Female	copd:Normal_Weight	copd:Stage4	copd:Absence_of_Risk
BB	copd:non-Cachetic	copd:Normal_Weight	copd:Stage4	copd:Absence_of_Risk
BB	copd:non-Sarcopenic	copd:Normal_Weight	copd:Stage4	copd:Absence_of_Risk
CV	copd:Male	copd:Obesity_1st_Degree	copd:Stage3	copd:Mild_Risk
CV	copd:non-Cachetic	copd:Obesity_1st_Degree	copd:Stage3	copd:Mild_Risk
CV	copd:non-Sarcopenic	copd:Obesity_1st_Degree	copd:Stage3	copd:Mild_Risk
...	...	...	...	...

^a^Data not reported for conciseness.

#### Ontology Use and Updating

The COPD and Nutrition ontology described in the previous subsection is a tested prototype able to classify patients properly and provide clinicians with nutrition-related recommendations. To support clinical personnel, the ontology-based DSS needs to be integrated into digital applications, enabling clinicians to access its functionalities intuitively and easily (as described in the following section). This would also enable the assessment of the usefulness and accuracy of the inferences by leveraging on clinicians outside the development team.

### The Application for Clinical Personnel

The clinician application was developed to run on a Windows PC or laptop and allows the lung specialist to obtain an overview of the patient’s nutritional condition and generate tailored dietary recommendations. To achieve this, the application is connected to the DSS (hosted on a semantic repository) with permission to modify the ontology, reason over new input data, and obtain a new set of dietary recommendations. The application and DSS communication are based on SPARQL queries running over the Stardog reasoner. The entire architecture has already been tested in previous work [24,63].

**Figure 3:**
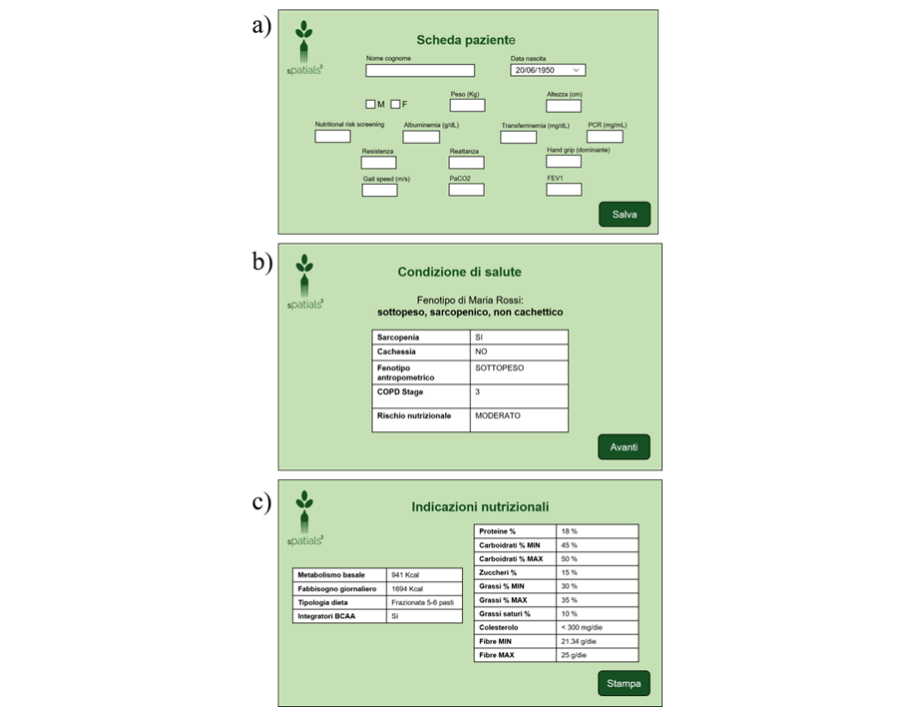
The 3 main graphical user interface panels of the clinician application: (A) patient profile, (B) health condition, and (C) nutritional recommendations.

The application is based on a simple and intuitive graphical user interface (GUI). Its flow is as follows:

1. The clinician logs in to the application either to create a new patient profile or to modify an existing one. The Patient Profile (Scheda Paziente) panel (Figure 3A) has several fields corresponding to the input information needed by the DSS to represent the user’s health condition. These include personal data and the results of the patient assessment.
2. Once the new patient profile is saved, the application sends a SPARQL query INSERT to upload the new information into the ontology. The DSS reasons over the new data to obtain the patient’s classification and the nutritional recommendations. The output is sent back to the application in the form of a JSON file to populate the GUI panels.
3. The Health Condition (Condizione di Salute) panel, shown in Figure 3B, shows the classification results: metabolic phenotype, presence of sarcopenia and cachexia, anthropometric phenotype, COPD stage, and NRI.
4. The subsequent panel, the Nutritional Recommendations (Indicazioni Nutrizionali), shown in Figure 3C, summarizes the nutritional recommendations, indicating basal metabolism, daily intake, type of diet, suggested BCAA supplement, percentage, and grams of micro- and macronutrients.
5. The clinician can save the recommendations in a PDF file or print them to share them with the dietician to support them in preparing a diet for the patient.

### Preliminary Validation With Clinical Personnel

#### Overview

Before the implementation in a real use-case scenario, we performed an expert validation of the DSS as a preliminary but necessary step. In total, 2 experiments were performed—considering the multidisciplinary nature of the tool, it was necessary to validate 2 aspects of the DSS with 2 different groups of clinicians.

Therefore, each experiment was carried out by a specific group of clinical experts: (1) a group of nutrition experts validated the recommendations generated by the DSS, and (2) a group of specialists in respiratory diseases evaluated the acceptability of the digital application in clinical practice based on the COPD DSS.

#### Procedure

Participants were recruited through email invitations among the national experts in nutrition and pulmonology, identified by searching the literature and professional networks. Once they expressed willingness to participate in the study, they signed a written informed consent form and agreed to the data treatment according to the General Data Protection Regulation. In total, 2 experimenters scheduled the web-based video calls—one for each participant—between November 2022 and January 2023. During the test, the experimenters briefly introduced the aim of the system and the main steps of the validation and recorded the participants’ answers to brief ad hoc questionnaires and spontaneous comments. Descriptive statistics were calculated for each variable, and the spontaneous comments were analyzed and categorized based on their content.

#### Experiment 1: Validation of the DSS Recommendations

The first experiment focused on validating the nutritional recommendations generated by the DSS. A total of 7 nutrition experts participated in the validation. The experimenters showed the participants the profiles of 5 real patients and the inferred recommendations. The patient profiles were obtained from real clinical cases provided by the clinicians involved in the project. Each profile included the patient ID, age, gender, and health condition containing all the clinical parameters needed as input to the system.

**Figure 4:**
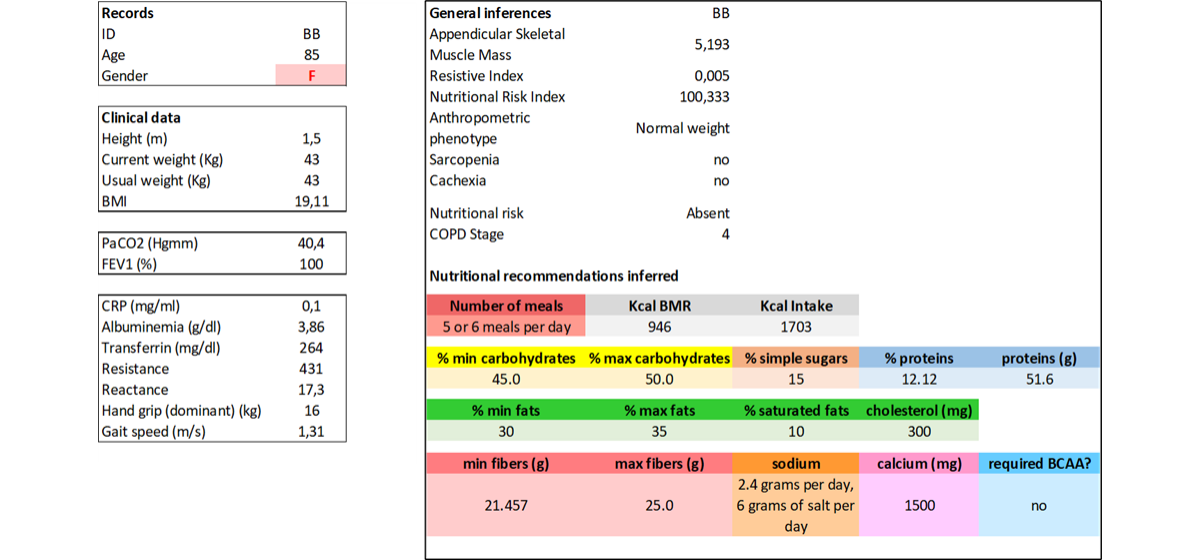
An example of a patient profile provided to clinicians (on the left) and the inferences drawn by the ontology-based decision support system (on the right).

After presenting the patient’s condition, the experimenter showed the recommendations generated by the DSS, which included the patient’s inferred classification (metabolic phenotype, presence of sarcopenia or cachexia, anthropometric phenotype, COPD stage, and NRI) and the nutritional recommendations with information on metabolism and suggested quantities of macro- and micronutrients. An example of a patient profile used during the experiment is presented in Figure 4, whereas all the patient profiles used during the evaluation are available in Multimedia Appendix 3. Participants were granted up to 15 minutes to observe the presented patient’s health condition. During this time, participants were free to perform calculations using the data shown, ask questions (if necessary) to the experimenter, and consult external sources (eg, books and papers). After the 15-minute period, for each patient, we asked the participants to rate on a scale from 1 to 5 how much they agreed with the recommendation; in case of a score of <5, we asked the participant to provide a brief explanation. We also collected spontaneous comments that emerged during the experiment. The maximum duration of the experiment for each participant was 1 hour and 15 minutes.

#### Experiment 2: Acceptability Evaluation

**Figure 5:**
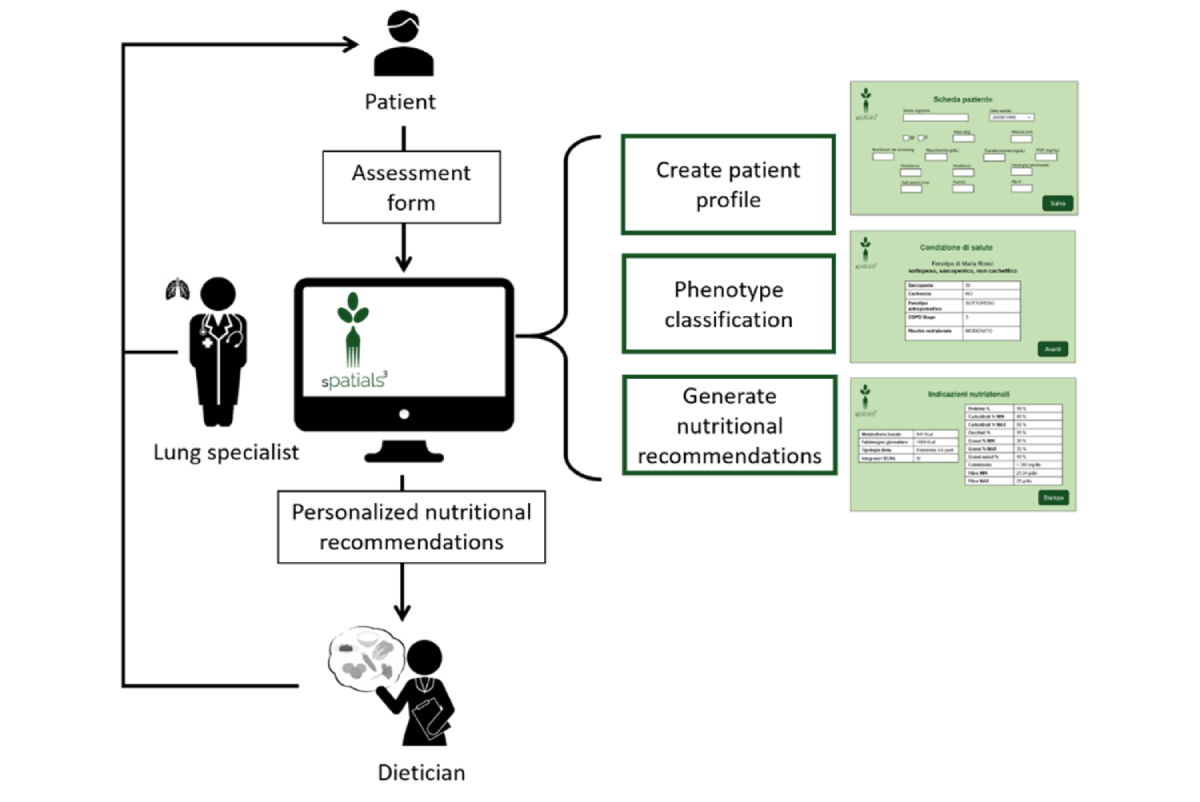
The process of use of the chronic obstructive pulmonary disease decision support system allowing the professionals to generate nutritional recommendations for a specific patient and some screenshots of the main graphical user interface panels (patient profile, patient classification, and nutritional recommendations).

The second evaluation focused on assessing the overall system acceptability, including the DSS and a digital application working as a GUI for inserting patient data and retrieving personalized recommendations. A total of 7 lung specialists agreed to participate in the experiment. The experimenter explained to each participant the expected process of use of the system and the application data flow in a daily clinical routine, as summarized in Figure 5. The lung specialists performed the patient assessment; inserted the clinical data to generate the patient profile on the application GUI, as described in the Application for Clinical Personnel section; and generated the nutritional recommendations**.**

Participants were granted 10 minutes to assess the application, ask the experimenters questions, or ask to be presented with the data flow again.

After this time, the experimenter administered an ad hoc questionnaire based on the subscales of the technology acceptance model by Davis [64] and its subsequent amendments [65] focused on perceived usefulness and intention to use. The participants had to rate on a scale from 1 to 5 the level of agreement with the following statements: (1) “I think that the proposed system is useful for clinicians”; (2) “I think that by using this application could enhance the treatment of individuals with COPD”; and (3) “if I had this application at work, I would use it.”

We also asked them to specify at least one reason why the application could be useful or not.

#### Results

##### Experiment 1 Results: Validation of the DSS Recommendations

A total of 7 experts (mean age 46.60, SD 13.35 years; n=7, 100% female) participated in the first validation phase. They were all dieticians with an average of 25.57 (SD 11.49) years of professional experience. The level of agreement with each recommendation is reported in Table 6 in terms of mean, SD, and minimum and maximum score.

The comments provided by each participant were analyzed and categorized according to their content. The categorization and frequency of the comments and the patient profiles that originated them are reported in Table 7.

**Table 6:** Validation—level of agreement scores (mean and SD) for each patient profile (BB, FG, LA, TM, and XY2 are the patient IDs) expressed by each participant; mean and SD for each patient profile.

	Scores (1-5)								Values, mean (SD)
BB	5	4	5	5	3	4	5	4.43 (0.79)	
FG	5	1	4	2	3	4	4	3.29 (1.38)	
LA	5	4	5	5	4	4	4	4.43 (0.53)	
TM	5	4	5	5	3	4	5	4.43 (0.79)	
XY2	5	4	5	5	4	4	5	4.57 (0.53)	

**Table 7:** The list of comments provided by clinicians categorized according to their content (category and comment) and linked to the patients that originated them (patient IDs); the frequency of each comment is reported in parentheses.

Category	Comment	Patient ID (frequency)
Quantity	“Too high-calorie uptake”	BB (1), FG (6), TM (1), and ANM (1)
Quantity	“Too high protein uptake”	FG (2) and LA (2)
Quantity	“Consider reducing simple sugars from 15 to 10%”	FG (1), LA (1), TM (2), and ANM (1)
Assessment	“I suggest including the physical activity level during the assessment and adjust correction factors accordingly”	All (2)

##### Experiment 2 Results: Acceptability Evaluation

A total of 7 experts (mean age 44.71, SD 11.94 years; n=7, 100% female) participated in the second validation phase. Of the 7 experts, 5 (71%) were lung specialists, 1 (14%) was a specialized lung physician, and 1 (14%) was a surgeon of the respiratory system. They had, on average, 14.73 (SD 9.67) years of professional experience, ranging from a minimum of 1 year for the specialized physician to 31 years. The acceptability score for the 3 subscales of perceived usefulness and intention to use was >4 points out of 5, as reported in Table 8.

Our system was considered useful for clinical practice because (1) it promotes the importance and facilitates the inclusion of the nutritional aspect in PR, as stated by 43% (3/7) of the participants; (2) it quickly provides a complete overview of the patient’s condition, according to 57% (4/7) of the participants; and (3) it fosters the multidisciplinary collaboration between lung specialists and dieticians. In addition, 86% (6/7) of the participants spontaneously commented on the ease of use of the application GUI that allowed the clinician to insert the patient assessment and obtain the nutritional recommendations.

**Table 8:** Acceptability—technology acceptance model subscale scores reported by each participant for perceived usefulness (PU1 and PU2) and intention to use (INT); mean and SD are reported for each subscale.

	Scores (1-5)								Values, mean (SD)
INT	4	4	4	5	4	4	4	4.86 (0.38)	
PU1	5	5	5	5	4	5	5	4.86 (0.38)	
PU2	5	5	5	5	5	5	4	4.14 (0.38)	

## Discussion

### Principal Findings

We performed 2 types of evaluation with 2 different experiments aimed at validating the nutritional recommendations generated by our system by nutrition experts and assessing the system’s acceptability by lung specialists. The first validation—performed by 7 nutrition experts—demonstrated that our system is able to provide meaningful and safe recommendations overall in compliance with clinical practice. In 80% (4/5) of the cases, the level of agreement between the human and the “digital” expert was approximately 4.5 points out of 5. In one case (patient FG), the experts did not completely agree with the recommendations, reporting a score of 3.29, which is not considered a disagreement. However, such a score was associated with the case of a critical patient who, in addition to COPD, had second-degree obesity. This is because our system is highly specialized in treating patients with COPD, who need a higher energy intake to cope with impaired respiratory functionality. In such critical cases, the active involvement of the clinician in the process becomes essential. For instance, the clinician may adjust the nutritional recommendations for the patient to lose weight while monitoring them. It is crucial that such a patient does not lose muscular mass instead of fat mass.

The comments were positive overall and could be considered more as suggestions than criticisms. The presence of small disagreements among experts (eg, regarding the calculation of the quantity of macro- and micronutrients) confirms the need for maintaining “the clinician in the loop,” as prescribed by the AgiSCOnt methodology adopted for the development of our DSS. In fact, although our system provides a useful and easy way of generating recommendations, each clinical case should be carefully considered, and slight modifications should be made by the clinician themselves in person.

The same considerations apply to the spontaneous comments, summarized in Table 7. The main concerns were about the percentage of simple sugars, which, for 4 patients, could be reduced from 15% to 10%. The guidelines indicate 15% as the maximum value, which should be adjusted by the clinician based on the percentage of other macronutrients. The other comments revealed a slight disagreement on the overall energy and protein intake, which was sometimes considered too high. However, our system is specifically focused on COPD; therefore, a higher intake was justified by the need to compensate for impaired respiratory function. Our group of experts was representative of the Italian clinical scenario, in which the influence of COPD on a patient’s nutrition is sometimes neglected. Therefore, such a result strengthens the rationale of our work, which provides a system able to help professionals and clinical care facilities, which often lack specialized services, identify particular needs toward more personalized and effective care.

The second evaluation demonstrated the acceptability of our system by a group of final users (ie, lung specialists involved daily in the assessment and therapy of patients with COPD). The usefulness of our system was confirmed and was especially related to the possibility of strengthening the consideration of nutritional aspects as part of PR standard practice. This is considered crucial by most specialists and the scientific community; however, due to organizational issues, it is not always considered [4]. Another crucial aspect that emerged was related to the importance of a multidisciplinary approach, and our system could especially help ease the cooperation between lung and nutrition specialists. At the same time, it could help lung professionals in extending their knowledge by considering aspects not strictly related to their expertise. Finally, all participants expressed their willingness to have such a system available in their daily clinical routine. They also considered it easy to use as the GUI was clear and the process was intuitive.

### Limitations

This work is not without limitations. First, our system was designed for the Italian context. The DSS is based on the national nutritional guidelines—it was necessary to follow a recognized standard, which may be different from one country to another. Similarly, the dietary recommendations are based on the Italian diet. This was necessary to provide a tool that can be effectively used by our target users (ie, lung specialists treating patients with COPD in the Italian health care system). The DSS’s modularity allows it to overcome such a limitation easily—the DSS could be adapted to include nutritional recommendations for other countries. The second limitation is about the participants of our validation experiments. The first experiment was based on the evaluation of 5 patient profiles by a group of nutrition experts. The number of patients examined was identified as the best compromise between a comprehensive representation of the clinical context and organizational aspects. Despite being few, the proposed patient profiles covered most of the potential real clinical cases. Regarding the acceptability evaluation, the main limitation resides in the fact that participants were homogeneous in terms of age (most of them were aged 45-50 years), culture, geographical location, and language (all of them were Italian). Such sociocultural factors are known to impact digital health technology use [66]; however, as previously stated, our work at this stage is focused on the Italian scenario, and therefore, our sample can be considered representative of the final population of target users.

### Future Work

As recently noted, most digital health applications remain limited to pilot studies—mainly because they fail in the proposed aims or face significant implementational barriers [67]. From a digital health application perspective, our experiments aimed to verify the stability of the developed solution—in line with the WHO’s guidelines [23]. In particular, the validations verified the performance consistency, the proposed solution’s overall feasibility, and the digital tool’s efficacy. Considering the early stage of the DSS and its application, we need to further investigate the implementation protocols and to acquire long-term proof of the efficacy of the tool among pneumologists and patients with COPD (ie, the acceptability of the tool needs to be verified with a larger sample of end users and in a real clinical setting so that more pneumologists can provide feedback regarding the tool’s perceived usefulness, ease of use, and satisfaction; moreover, the effectiveness of the proposed diets should be tested with patients with COPD). To achieve these objectives, more extensive experimentation with larger samples of participants (clinical nutritionists, dieticians, and pneumologists) is necessary. Moreover, the involvement of clinical personnel can support the identification of implementation protocols suitable for the adoption of the digital tool in clinical practice. In this way, the application’s level of maturity could move from early to mild (according to the WHO [23]), where its effectiveness can be tested in a nonresearch (uncontrolled) setting.

To support the prompt identification of barriers and implementational challenges, reporting the development of the COPD DSS within a framework for the definition of digital health application implementation can be useful. In particular, the Guidelines and Checklist for the Reporting on Digital Health Implementations [67], by providing a list of 20 items to be monitored, can foster the identification of issues in the Technical design phase in the Interoperability and Data management areas.

In this regard, the availability of data and the implementation of the application within the health system are another relevant node to be investigated. Although the current version of the digital application is still in its prototypical phase, scaling up the application to the regional or national level (ie, *coverage* in the Guidelines and Checklist for the Reporting on Digital Health Implementations) is essential to ensure its use in clinical practice. Therefore, toward this aim, strategies for collecting the outputs and making them available in patients’ data (or electronic health records) need to be investigated. In this regard, scientific literature offers some interesting approaches grounded in the Italian health care system that could be considered to make the COPD DSS interoperable with existing tools [68-70]. As the COPD DSS leverages ontologies to represent its data, this technology can be used to achieve semantic interoperability of the information [68], moving a step toward the longitudinal collection of health data about patients [70] while ensuring data protection according to the national and European laws [69].

Finally, from an ontological perspective, the domain ontology regarding COPD presented in this work could benefit from mapping with existing (and larger) biomedical standard ontologies to increase its shareability (eg, the WHO’s International Classification of Diseases and International Classification of Functioning, Disability and Health, as well as upper biomedical ontologies)—a best practice of ontology engineering [71]. Moreover, considering that the proposed system can be adapted to any other national clinical context by modifying the domain ontologies, a possible future research direction consists of involving international clinicians to increase the knowledge formalized in the ontologies so that it is possible for the DSS to cover the specific nutritional indications of different countries.

### Conclusions

The role of nutrition in the management of patients with COPD is often underestimated, although scientific evidence points toward the important role that diet plays in PR. The nutritional status of patients with COPD is essential to prevent exacerbations and avoid comorbidities, but attention to the patient’s body composition and nutritional status is often secondary in clinical practice. This may be partially because lung specialists may lack specialized training in clinical nutrition, although they recognize the relevance of dietary recommendations in PR.

An ontology-based DSS was developed to support pneumologists in considering nutritional aspects. The DSS formalizes expert knowledge in computable models able to infer patient-tailored nutritional recommendations, leveraging a set of information to capture the nutritional and physical status of the patient; therefore, by applying rules, it can support the classification of patients with COPD and provide tailored recommendations indicating the percentages and amounts of micro- and macronutrients that should make up their diet. The domain ontologies act as the backbone of a clinician-dedicated application.

The application was validated to assess the clinical compliance of the DSS’s recommendations and the acceptability of such an application in clinical practice by lung specialists. For both validations, the proposed system performed more than adequately—in particular, pneumologists underlined the role that such an application may play in achieving a multidisciplinary approach in PR. This paper concludes by investigating future research directions to implement the COPD DSS further into a fully-fledged digital health application.

## Appendix

Multimedia Appendix 1 A table representing the set of rules for providing phenotype-tailored recommendations.

Multimedia Appendix 2 The chronic obstructive pulmonary disease decision support system domain ontology.

Multimedia Appendix 3 A table reporting the nutritional recommendations for each patient used to test the validity of the ontology rule set.

## Abbreviations

AgiSCOnt: Agile, Simplified, and Collaborative Ontology Engineering Methodology
BCAA: branched-chain amino acid
BMR: basal metabolic rate
COPD: chronic obstructive pulmonary disease
CQ: competency question
DSS: decision support system
FEV1: forced expiratory volume in the first second
FFM: fat-free mass
GUI: graphical user interface
LARN: Livelli di Assunzione di Riferimento di Nutrienti ed energia
NRI: nutritional risk index
PR: pulmonary rehabilitation
SWRL: semantic web rule language
WHO: World Health Organization

## Data Availability

All data generated or analyzed during this study are included in this published paper.
