# Supplementary material for: An Ontology-Based Decision Support System for Tailored Clinical Nutrition Recommendations for Patients With Chronic Obstructive Pulmonary Disease: Development and Acceptability Study
Source: JMIR Med Inform. 2024 Jun 26;12:e50980. doi: 10.2196/50980 (PMC11237782; doi:10.2196/50980)
Supplement: Multimedia Appendix 1 [file medinform_v12i1e50980_app1.docx]

**Appendix**

Appendix 1. A table representing the set of rules for providing phenotype-tailored recommendations.
