# Supplementary material for: An Ontology-Based Decision Support System for Tailored Clinical Nutrition Recommendations for Patients With Chronic Obstructive Pulmonary Disease: Development and Acceptability Study
Source: JMIR Med Inform. 2024 Jun 26;12:e50980. doi: 10.2196/50980 (PMC11237782; doi:10.2196/50980)
Supplement: Multimedia Appendix 3 [file medinform_v12i1e50980_app3.docx]

| **?id** | **?rec** | **?bmr** | **?kcalInt** | **?prot** | **?minCar** | **?maxCar** | **?sugar** | **?minFat** | **?maxFat** | **?satFat** | **?col** | **?minFib** | **?maxFib** | **?bcaa** |
| --- | --- | --- | --- | --- | --- | --- | --- | --- | --- | --- | --- | --- | --- | --- |
| 001 | copd:Rec_Pat_XY1 | 1010 | 1818 | 25.0 | 45.0 | 50.0 | 14.9 | 35 | 50 | 9.9 | 300 | 22.90 | 25.0 | yes |
| BB | copd:Rec_Pat_BB | 946 | 1703 | 12.11 | 45.0 | 50.0 | 14.9 | 30 | 35 | 9.9 | 300 | 21.45 | 25.0 | no |
| CV | copd:Rec_Pat_CV | 1590 | 2862 | 14.79 | 45.0 | 50.0 | 14.9 | 35 | 50 | 9.9 | 300 | 25.0 | 25.0 | only if does not reach protein intake with meals |
| DMP | copd:Rec_Pat_DMP | 1498 | 2696 | 14.78 | 45.0 | 50.0 | 14.9 | 30 | 35 | 9.9 | 300 | 25.0 | 25.0 | only if does not reach protein intake with meals |
| FA | copd:Rec_Pat_FA | 947 | 1421 | 19.45 | 45.0 | 50.0 | 14.9 | 30 | 35 | 9.9 | 300 | 17.90 | 25.0 | no |
| FG | copd:Rec_Pat_FG | 2081 | 3122 | 15.05 | 45.0 | 50.0 | 14.9 | 35 | 50 | 9.9 | 300 | 25.0 | 25.0 | only if does not reach protein intake with meals |

**Appendix**

Appendix 2. A table reporting the nutritional recommendations for each patient used to test the validity of the ontology rule set.
